# Neuregulin expression in solid tumors: Prognostic value and predictive role to anti-HER3 therapies

**DOI:** 10.18632/oncotarget.8648

**Published:** 2016-04-08

**Authors:** Alberto Ocaña, Laura Díez-González, Azucena Esparís-Ogando, Juan Carlos Montero, Eitan Amir, Atanasio Pandiella

**Affiliations:** ^1^ Translational Research Unit, Albacete University Hospital, Albacete, Spain; ^2^ Division of Medical Oncology and Hematology, Princess Margaret Cancer Centre, University of Toronto, Toronto, Canada; ^3^ Cancer Research Center (CIC-IBMCC), CSIC-University of Salamanca, Salamanca, Spain; ^4^ IBSAL, Salamanca, Spain

**Keywords:** neuregulin, anti-HER3, prognostic value, predictive role

## Abstract

**Background:**

Neuregulins (NRG) are a family of epidermal growth factor ligands which act through binding to HER3 and HER4 receptors. NRGs are widely expressed in solid tumors. Their prognostic significance or their role as predictors of benefit from anti-HER3 therapy is not known.

**Results:**

Of 29 included studies, 7 studies reported the association between NRG and outcome. NRG was most commonly expressed in breast, prostate, colon and bladder cancers. NRG expression was not associated with either OS or PFS (HR: 3.47, 95% CI 0.78–15.47, *p* = 0.10 and HR: 1.64, 95% CI 0.94–2.86, *p* = 0.08, respectively). In 4 placebo controlled trials of anti-HER3 therapy, the addition of anti-HER3 antibodies to control therapy in unselected patients was not associated with improved PFS (HR: 0.88, 95% CI 0.75–1.04. *p* = 0.14). However, in patients with high NRG expression, there was significantly delayed progression (HR: 0.35, 95% CI 0.23–0.52, *p* < 0.001). Anti-HER3 antibodies were associated with increased risk of diarrhea, nausea and rash.

**Methods:**

A search of electronically available databases identified studies exploring clinical outcomes based on NRG expression, as well as placebo-controlled trials of HER3-directed therapy reporting results based on NRG expression status. Data were combined in a meta-analysis using generic inverse variance and random effects modeling for studies reporting the hazard ratio (HR) for overall (OS) or progression-free survival (PFS). Mantel-Haenszel random-effect modeling was used for odds ratio (OR) for 3-year and 5-year OS and PFS.

**Conclusions:**

NRG expression is not associated with either OS or PFS, but is a predictor of benefit from anti-HER3 antibodies.

## INTRODUCTION

Neuregulins or Heregulins (NRG) are a family of the Epidermal Growth Factor (EGF) ligands that are widely expressed in solid tumors [1–3]. Four different genes named *NRG-1, NRG-2, NRG-3* and *NRG-4* code for more than to 32 different NRG isoforms [2]. The NRGs act by binding to the ErbB/HER family of receptor tyrosine kinases. Four different ErbB/HER receptors have been described in mammals: ErbB1/HER1/EGFR, ErbB2/HER2/neu, ErbB3/HER3 and ErbB4/HER4 [4, 5]. HER3 is the major NRG receptor [3, 5–7].

ErbB/HER receptors and their ligands have been widely studied in cancer and linked to oncogenic transformation [4]. They have also been the target for directed therapies, including monoclonal antibodies such as trastuzumab or pertuzumab against HER2, or cetuximab against EGFR; or tyrosine kinase inhibitors such as lapatinib against EGFR and HER2 [8]. Of note, therapeutic inhibition of these receptors has been linked to clinical antitumor activity confirming the oncogenic role of these receptors in cancer [8]. HER3 expression has been associated with worse clinical outcome, and agents trying to neutralize its activity are in clinical development [9]. The fact that NRGs are the main activating ligands of HER3 suggests that tumors with high levels of NRG could be those that respond better to anti-HER3 therapies [10, 11].

In the current article we evaluated the expression and prognostic role of NRGs in solid tumors using publicly available data. We also studied the association of the expression of NRGs with clinical response to anti-HER3 antibodies. Finally we explored the toxicity associated with these anti-HER3 antibodies.

## RESULTS

### Expression of NRG in solid tumors

A total of 29 studies reported data on expression of NRG in solid tumors [12–40]. Characteristics of included studies are shown in Table 1. NRG was more studied in breast cancer (9 studies) and prostate cancer (4 studies) followed by colon and bladder cancer (3 studies for each tumor).

**Table 1:** Characteristics on included studies

Type of tumor		ID	NRG	Assay	Estimate of expression (sample size)
**Bladder cancer**		Forster 2011 [12]	NRG1α, and NRG1β	RT-PCR	Not described (*n* = 59)
		Amsellem-Ouazana 2006 [13]	NRG1, NRG2 and NRG3	RT-PCR	Not described (*n* = 73)
		Memon 2004 [14]	HRG1α, HRG1β, HRG2α, HRG2β, HRG3 and HRG4	RT-PCR	47% HRG1α, 49% HRG1β, 53% HRG2α, 42% HRG2β, 49% HRG3 and 34% HRG4 (*n* = 88)
**Breast cancer**	Breast cancer	Seoane 2015 [16]	NRG	Inmunohistochemistry	34% (*n* = 76)
	HER2 negative breast cancers	Haas 2009 [17]	HRG	Inmunohistochemistry	26% (*n* = 171)
	Breast cancer	de Alava 2007 [18]	NRG	Inmunohistochemistry	50% (*n* = 151)
	Pre-invasive ductal carcinoma *in situ* of the breast (DCIS)	Marshall 2006 [19]	HRG1α, HRG1β, HRG2α, HRG2β, NRG3 and NRG4	Inmunohistochemistry	30–80% (*n* = 60)
	Breast cancer	Dunn 2004 [20]	HRG1α, HRG1β, HRG2α, HRG2β, NRG3 and NRG4	Inmunohistochemistry	35–45% (*n* = 45)
	Locally advanced breast cancer	Raj 2001 [21]	NRG1	Inmunohistochemistry	84% (*n* = 115)
	Primary Breast Cancer	Esteva 2001 [22]	Heregulin	Inmunohistochemistry	48% (*n* = 35)
	Breast cancer	Visscher 1997 [23]	HRG	Inmunohistochemistry	38% (*n* = 34) and 50% (*n* = 34)*
	Breast cancer	Normanno 1995 [26]	HRG	Western blotting	25% (*n* = 60)
**Colon cancer**	Colorectal cancer	Mitsui 2014 [24]	HRG	Inmunohistochemistry	46% (*n* = 155) (cytoplasm of cancer cells)
	Colorectal cancer	Boeck 2012 [25]	tNRG1 (transmembrane neuregulin 1)	Inmunohistochemistry	76% (*n* = 54) (stromal)
	Colon cancer	Venkateswarlu 2002 [26]	Heregulin	Inmunohistochemistry	Not described
**Endometrial cancer**		Srinivasan 1999 [37]	NRG1α and NRG1β	Inmunohistochemistry	Not described (*n* = 41)
**Gastrointestinal malignant lymphoma**	7 mucosa-associated lymphoid tissue (MALT) lymphomas, 6 follicular lymphomas (FLs), 2 mantle lymphomas, 7 diffuse large B cell lymphomas (DLBCLs), 1 T cell lymphoma and 3 Burkitt lymphomas	Ebi 2011 [28]	NRG4	Inmunohistochemistry	48% (*n* = 26)
**Hepatocellular carcinoma (HCC)**		Hsieh 2011 [29]	NRG1	Immunoblotting analysis	100% (*n* = 9)
**Lung adenocarcinoma**		Pan 2015 [30]	NRG1	Inmunohistochemistry	49% tumor, 10% stroma (*n* = 115)
**Medulloblastoma**		Gilbertson 1998 [31]	NRG1β	Inmunohistochemistry	87% (*n* = 48)
**Oropharyngeal Squamous Cell Carcinoma (OPSCC)**		Qian 2015 [32]	HRG mRNA	*In situ* hybridization	77% (*n* = 96)
**Ovarian cancer**		Gilmour 2002 [33]	NRG1α and NRG1β	Inmunohistochemistry and RT-PCR	Inmunohistochemistry: 77% NRG1α - 87% NRG1β (*n* = 53) and RT-PCR: 83% NRG (*n* = 24)
**Pancreatic ductal adenocarcinoma (PDAC)**		Kolb 2007 [34]	HRG	Inmunohistochemistry	85% (*n* = 14)
**Papillary thyroid cancer**		Fluge 2000 [35]	HRG precursor	Inmunohistochemistry	78–83% (*n* = 134)
**Prostate cancer**	Prostate cancer	Hayes 2011 [36]	NRG4 (anti-127: all NRG4 isotypes, anti-123: NRG4α1 and NRG4α2, anti-128: NRG4α1, anti-135 : NRG4α2, anti-134: NRG4β3)	Inmunohistochemistry	Anti-123: weak (40%), moderate (45%), strong (17.5%); Anti-127: weak (45%), moderate (0%), strong (0%); Anti-128: weak (38.5%), moderate (12.8%), strong (2.5%); Anti-134: weak (46.2%), moderate (0%), strong (0%); Anti-135: weak (23.7%), moderate (7.9%), strong (0%) (*n* = 40)
	Adenocarcinoma prostate	Grimsley 2010 [37]	HRG	Inmunohistochemistry	Cytoplasm 99%, cell membrane 46%, nucleus 54% (*n* = 45)
	Prostate cancer	Lyne 1997 [38]		Inmunohistochemistry	100% (*n* = 24)*
	18 well, 15 moderately and 17 poorly differentiated	Leung 1997 [39]	HRGα	Inmunohistochemistry	72% (*n* = 50)
**Vestibular schawannoma**		Hansen 2004 [40]	NRG	Inmunohistochemistry	100% (*n* = 8)

### Association of NRG with clinical outcome

**Figure 1:**
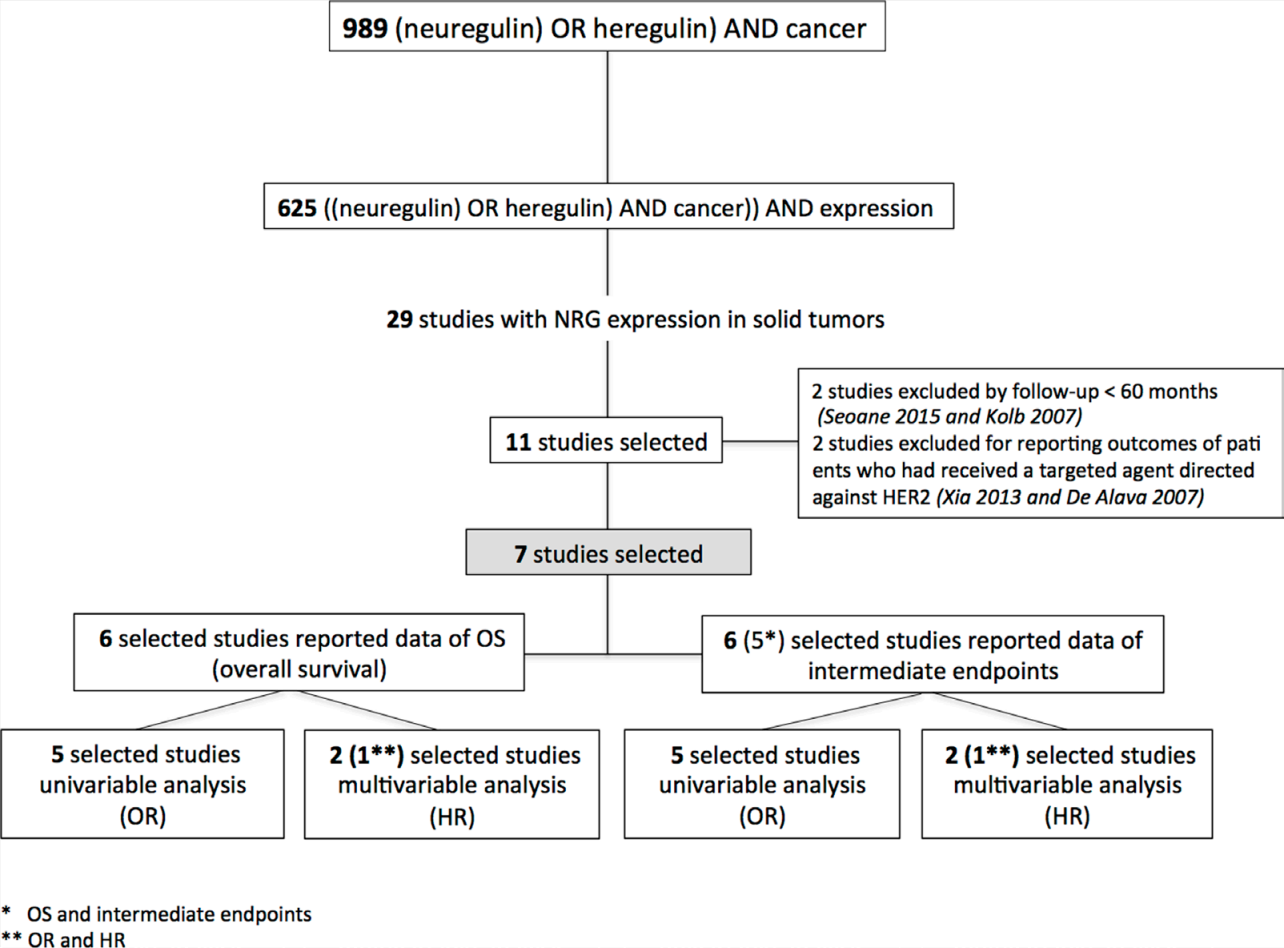
Flow chart for the selection of studies

A total of eleven studies reported the association between NRG and outcome. Seven were included in the analyses for the specific follow-up time points. Of these, six studies reported data on OS [14, 20, 24, 30, 32, 37] and six studies reported data on intermediate endpoints such as PFS or time to relapse [20, 24, 25, 30, 32, 37]. Figure 1 shows the flow chart for the selection of studies.

### Overall survival

**Figure 2:**
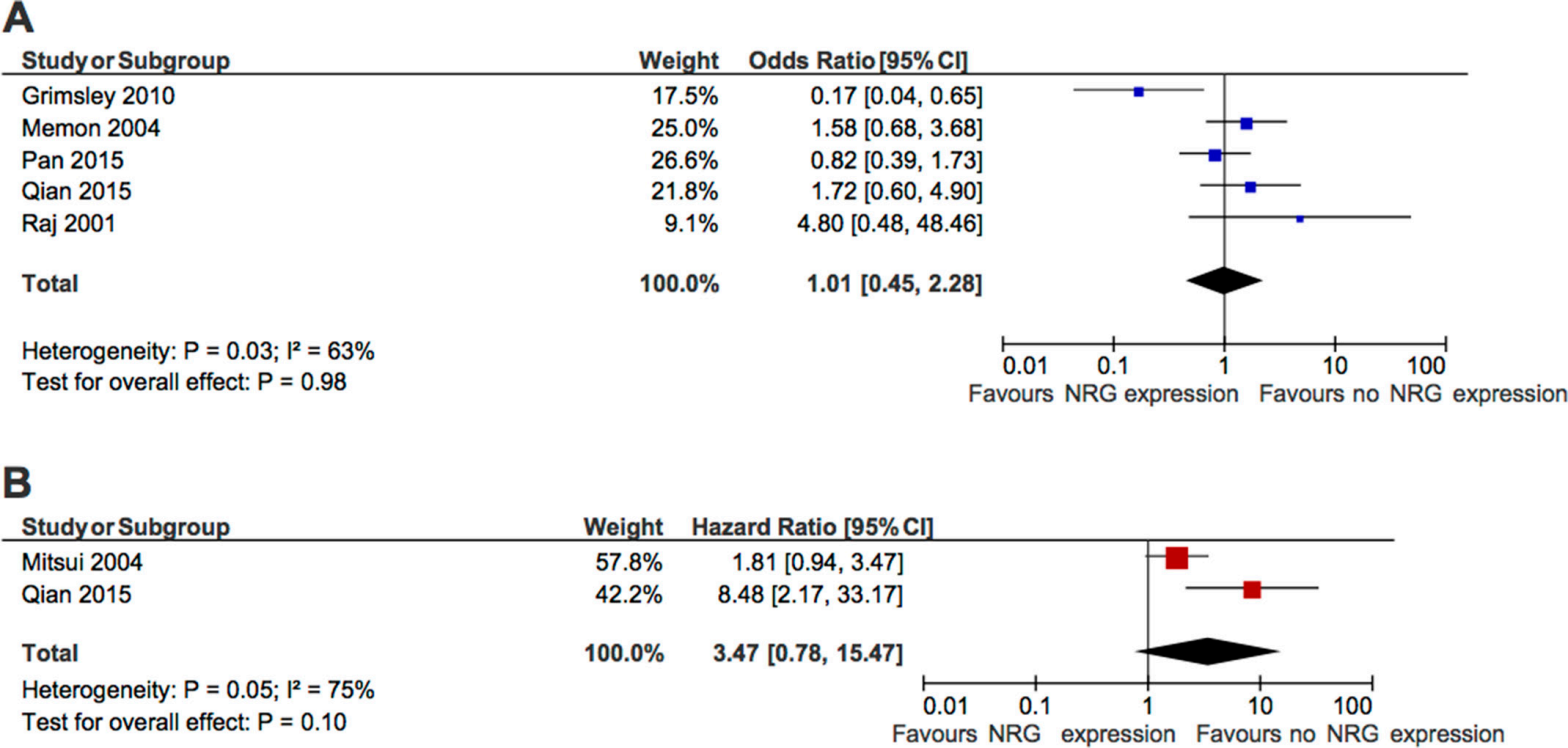
Forest plots showing association between NRG expression and overall survival (**A**) Odd of survival at 5 years. (**B**) Hazard ratio for survival.

When all studies were pooled, there was no apparent association between NRG and OS (OR for 5 year OS: 1.01, 95% CI 0.45–2.28, *p* = 0.98, Figure 2A). There was significant heterogeneity (Cochran Q *p* = 0.03, *I*^2^ = 63%) with one study in prostate cancer [37] showing an association with improved outcomes while the remaining individual studies showed no significant association. Exclusion of the outlying study did not change the results significantly (OR for 5 year OS: 1.30, 95% CI 0.78–2.14, *p* = 0.31). Similar results were observed in the two studies [24, 32] that reported HR for OS (pooled HR: 3.47, 95% CI 0.78–15.47, *p* = 0.10, Figure 2B) and when OS was examined at 3 years (pooled OR: 1.25, 95% CI 0.80–1.95, *p* = 0.33).

### Progression-free survival

**Figure 3:**
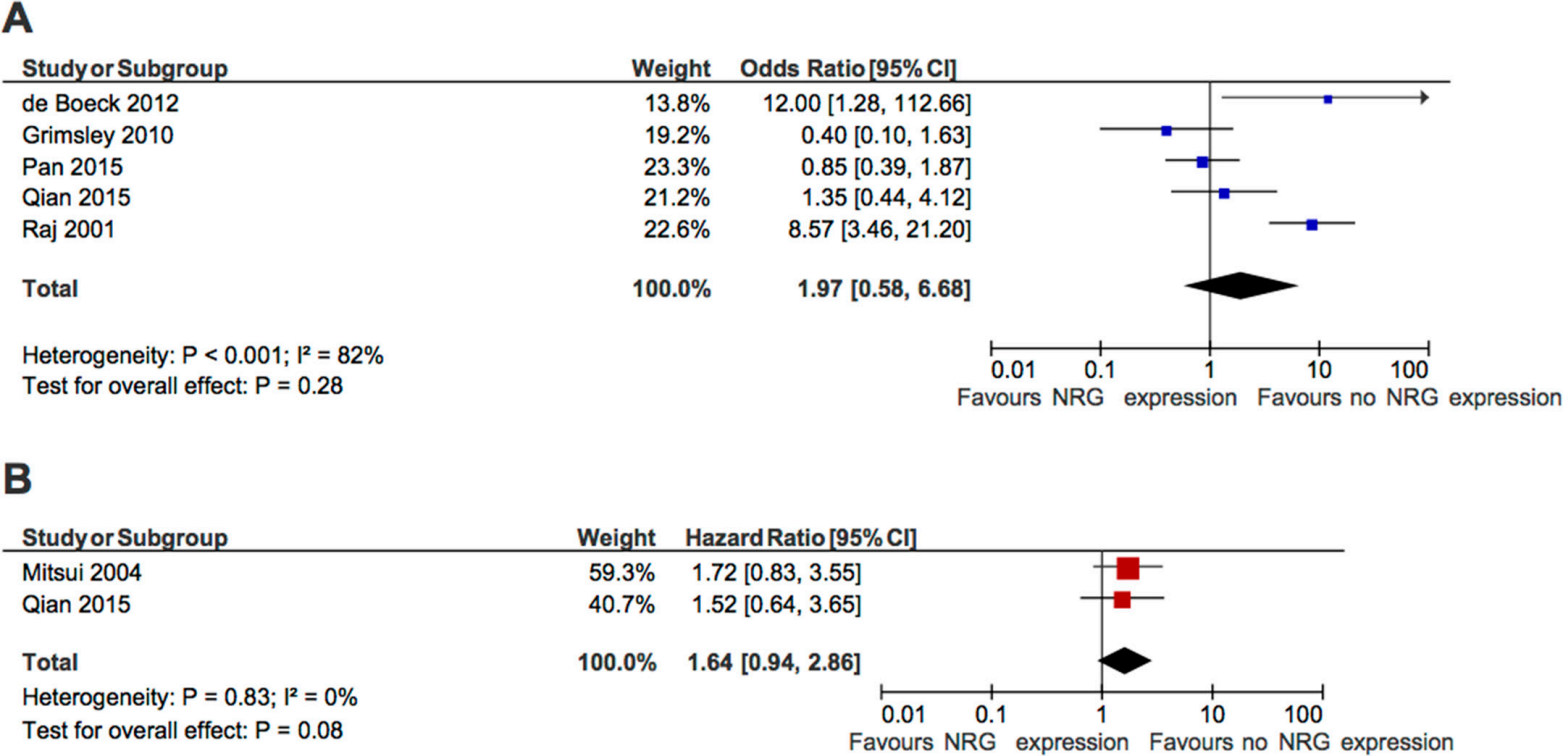
Forest plots showing association between NRG expression and progression-free survival (**A**) Odd of freedom from progression at 5 years. (**B**) Hazard ratio for progression-free survival.

When all studies were pooled, there was no apparent association between NRG and PFS (OR for 5 year PFS: 1.97, 95% CI 0.58–6.68, *p* = 0.27, Figure 3A). Once again, there was significant heterogeneity (Cochran Q *p* < 0.001, *I*^2^ = 82%), although for PFS caused by general heterogeneity and not individual outlying studies. Similar results were observed in the two studies [24, 32] that reported HR for PFS (pooled HR: 1.64, 95% CI 0.94–2.86, *p* = 0.08, Figure 3B) and when PFS was examined at 3 years (pooled OR: 2.17, 95% CI 0.76–6.21, *p* = 0.15).

### NRG and benefit from anti-HER3 therapies

**Figure 4:**
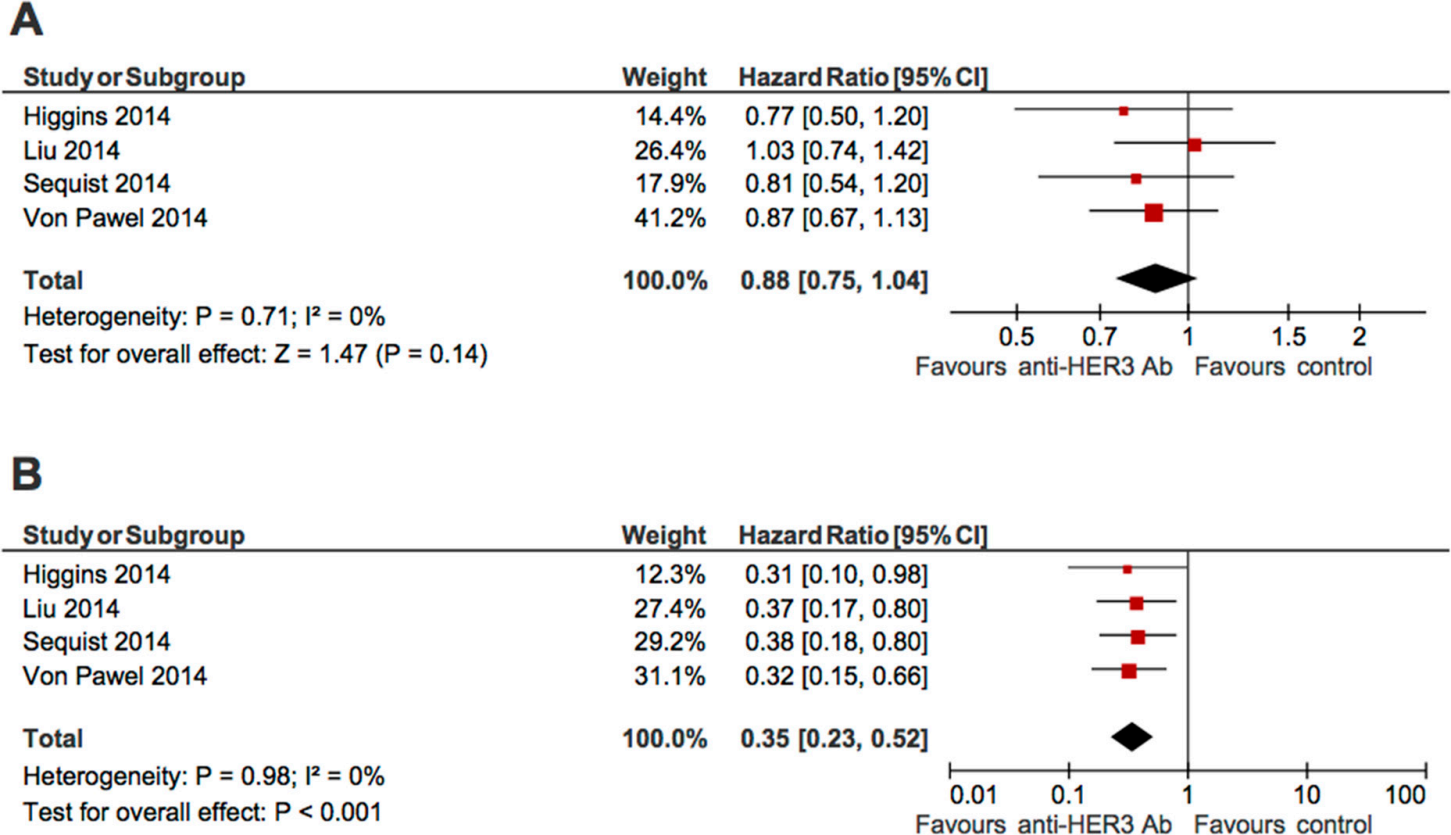
Forest plots showing effect of anti-HER3 antibodies on progression-free survival among unselected patients (A), or patients with NRG expression (B)

Next we aimed to explore if the expression of NRG was a predictor of benefit from therapy with anti-HER3 antibodies. To do so, we pooled the HR for PFS among four randomized trials [41–44] (Table 2). Results showed that in patients unselected for NRG, anti-HER3 antibodies were not associated with improved PFS (pooled HR: 0.88, 95% CI 0.75–1.04. *p* = 0.14, Figure 4A). However, among patients with NRG expression, there was significantly delayed progression (pooled HR: 0.35, 95% CI 0.23–0.52, *p* < 0.001, Figure 4B).

**Table 2:** Characteristics of ongoing studies with anti-HER3 antibodies

ID	Poster Title	Congress	Total (*n*)	Type tumor	Determination of NRG
Higgins 2014 [41]	A randomized, double-blind phase II trial of exemestane plus MM-121 (a monoclonal antibody targeting ErbB3) or placebo in postmenopausal women with locally advanced or metastatic ER+/PR+, HER2-negative breast cancer	2014 ASCO Annual Meeting	115	Locally advanced or metastatic ER+/PR+, HER2-negative breast cancer	BM+ patients were defined as having: • High HRG mRNA by RT-PCR (score > −5) • Low ErbB2 by qIHC (Log_10_ ErbB2 < 5.1)
Liu 2014 [42]	A phase II randomized open-label study of MM-121, a fully human monoclonal antibody targeting ErbB3, in combination with weekly paclitaxel versus weekly paclitaxel in patients with platinum-resistant/refractory ovarian cancers	2014 ASCO Annual Meeting	223	Platinum resistant/refractory ovarian cancers	BM+ patients were defined as having: • Detectable HRG mRNA by RNA-ISH • Log_10_ ErbB2 < 5.1 by qIHC
Sequis 2014 [43]	A randomized phase 2 trial of MM-121, a fully human monoclonal antibody targeting ErbB3, in combination with erlotinib in EGFR wild-type NSCLC patients	2014 ASCO Annual Meeting	129	EGFR wide-type NSCLC	BM+ patients were defined as having detectable HRG mRNA by RNA-ISH
Von Pawel 2014 [44]	Phase 2 HERALD study of patritumab with erlotinib in advanced NSCLC subjects	2014 ASCO Annual Meeting	141	Advanced NSCLC (high dose) ITT population (intent-to-treat)	• A validated quantitative polymerase chain reaction assay was developed to measure mRNA in formalin-fixed paraffin-embedded tissue. • The HRG cutoff was set at the median delta threshold cycle (ΔCt) based on samples that were blinded with respect to treatment group and clinical outcomes

### Toxicity of anti-HER3 therapies

Anti-HER3 antibodies were associated with significantly increased odds of diarrhea, nausea and vomiting, and rash as shown in Table 3.

**Table 3:** Pooled analyses of toxicities from anti-HER3 antibodies

Toxicity	Pooled OR	95% CI	*p*
Diarrhea	3.06	2.21–4.25	< 0.001
Nausea and vomiting	1.81	1.21–2.70	0.004
Rash	1.81	1.24–2.65	0.002

### Evaluation of NRG expression

Finally, we analyzed the different approaches used to evaluate the expression of NRG in solid tumors. In our retrospective analyses we observed that in the majority of studies the methods used were immunohistochemical assessment or polymerase chain reaction (PCR). Of note in those clinical studies in which the expression of NRG was used as a biomarker to select responsive patients, NRG was evaluated exclusively using mRNA by PCR (Table 2).

## DISCUSSION

In the present article we describe the prognostic role of NRG expression, and the predictive accuracy of NRG expression as a biomarker of benefit from anti-HER3 therapies. With respect to the first aspect, no clear association was found between NRG expression and clinical outcome when analyzing all the studies globally. However, patients with tumors that expressed high levels of NRG had significantly delayed progression of the disease when treated with anti-HER3 antibodies, compared with those without such expression.

Through binding to ErbB/HER receptors, particularly HER3, the NRGs control several biological responses linked to the malignant phenotype, including proliferation or metastatic dissemination [15, 45]. In this context, it was expected that tumors with high expression of this ligand were associated with poor outcome. In fact, for some tumor subtypes such as breast cancer, expression of specific isoforms of NRG have been linked with worse outcome [46]. However, when analyzing the overall relevance of NRG expression in several tumor types, this hypothesis was not verified suggesting that the role of NRG in cancer probably depends on other biological characteristics including tumor subtype. Moreover, since the actions of NRGs depend on the presence of their cognate receptors, the mere expression of NRGs may not be biologically fruitful unless expression of those receptors is present in the tumoral tissue. In fact, the link among patient outcome and expression of HER3 points in that direction. It is therefore likely that tumors expressing such receptor may be fed by NRGs produced by either the tumoral cells or their microenvironment. This biological situation may therefore be highly sensitive to agents, such as anti-HER3 antibodies, that disrupt the NRG-HER3 interaction and signaling axis. In line with this hypothesis is the fact that activation of HER3 by NRG in cellular models predicts response to anti-HER3 therapies or antibodies against HER2 [10, 17, 45]. This situation represented the basis for the selection of NRG as a biomarker of response to anti-HER3 strategies. Our pooled analyses validate results from individual studies, confirming its potential use as a biomarker of benefit from therapies using anti-HER3 antibodies.

Use of NRG as a biomarker for the selection of patients that may benefit from therapies based on anti-HER3 antibodies requires the development of a reliable test to measure NRG expression in tumoral samples. While in all ongoing studies testing anti-HER3 antibodies NRG has been evaluated by PCR, in the retrospective series this ligand was also studied using immunohistochemistry. A limitation of this approach is the existence of different isoforms of NRG, so it is mandatory to establish the isoforms present in the samples to be analyzed using clinically-friendly methods for their identification and measurement. Finally, the optimal cut-offs for defining positive expression in different tumors also require standardization.

This study has limitations. It is a study based on published data, so it could have a potential bias for the identification of only positive published studies. Secondly, as mentioned, the methods for identification of NRG expression were variable with some studies using antibodies and others selecting patients based on the expression of NRG mRNA by PCR. Finally the combination of different tumour types adds heterogeneity which may mask a true effect in a specific tumour type. This is a major limitation of the actual study. It will be desirable to reevaluate the relevance of NRG expression in the different tumors when more studies will be available.

In conclusion, this study suggests that assessment of NRG expression, despite showing no significant prognostic association with OS or PFS, is a predictor of benefit from anti-HER3 antibodies.

## MATERIALS AND METHODS

Preferred Reporting Items for Systematic Reviews and Meta-Analyses guidelines was used to guide this analyses [47].

### Data sources and study selection

Medline (Host: PubMed) was searched for studies published between September 1995 and October 2015, which evaluated the expression of neuregulin/heregulin in solid tumors by immunohistochemistry (IHC) or quantitative real-time RT-PCR. Studies using other assays were excluded to maintain homogeneity. We used the MeSHterms “neuregulin” or “heregulin” and “cancer”, adding the limitation of publications in English. Additional studies were identified through citation lists.

Two independent searches were conducted. First, we explored the association of NRG with clinical outcome. Eligible studies reported hazard ratios (HR) and 95% confidence interval (CI) and/or *p*-value for overall survival (OS) from multivariable analyses; or provided Kaplan-Meier curves for OS at 3 and 5 years based on the expression of NRG. Studies reporting outcome of patients who had received a targeted agent directed against HER2 were excluded as well as studies not reported as a final publication. Data for OS were preferred but if not available, studies reporting data on intermediate endpoints such as progression-free survival (PFS) or time to relapse were included and analyzed separately. For the purpose of this analysis, PFS and time to relapse were considered to be interchangeable. Second, we explored if the expression of NRG was a predictor of benefit of treatment with anti-HER3 antibodies. Eligible studies reported HR and 95% CI and/or *p*-value for PFS from multivariable analyses in placebo-controlled randomized trials of anti-HER3 agents. In this cohort of studies we also explored toxicities of anti-HER3 therapies.

### Data extraction

Two reviewers (LD, AO) evaluated independently all the titles identified by the search strategy. The results were then pooled and all potentially relevant publications retrieved in full and assessed for eligibility. Disagreement was resolved by consensus.

The following information was captured using data abstraction forms: Name of first author, year of publication, type of tumor, NRG studied, methods used for the evaluation of NRG, proportion of patients with NRG expression and the number of patients treated with anti-HER3 therapies. Survival data were estimated from multivariable analyses independently by two authors (EA, LD) and disagreement was resolved by consensus.

If HRs were not reported we extracted the odds of survival at three and five years from Kaplan-Meier curves and calculated odds ratios (OR) with 95% CI. For studies reporting both HR and Kaplan-Meier curves, we preferentially used the multivariable HR. Finally, we extracted data of the most commonly reported toxicities and calculated OR with 95% CI for these toxicities, comparing anti-HER3 therapies to placebo.

### Data synthesis and statistical analyses

Study characteristics were reported descriptively using means and proportions. Studies reporting HR for OS or PFS were weighted and pooled using the generic inverse variance and random-effect model [48]. Studies reporting the odds of death or progression at 3 or 5 years or the odds of commonly reported toxicities were weighted and pooled using the Mantel-Haenszel random-effect model. All meta-analyses were conducted using RevMan 5.3 analysis software (Cochrane Collaboration, Copenhagen, Denmark).

Statistical heterogeneity was assessed using the Cochran's Q and *I*^2^ statistics. Subgroup analyses were conducted as described by Deeks et al. [49]. All statistical tests were two-sided, and statistical significance was defined as *p* < 0.05. No corrections were made for multiple testing.
